# Supplementary material for: Comparative efficacy of tenofovir and entecavir in nucleos(t)ide analogue-naive chronic hepatitis B: A systematic review and meta-analysis
Source: PLoS One. 2019 Nov 21;14(11):e0224773. doi: 10.1371/journal.pone.0224773 (PMC6872143; doi:10.1371/journal.pone.0224773)
Supplement: Attachment — Submitted filename: Response to revierers.pdf [file pone.0224773.s002.pdf]

Dear Academic Editor,

On behalf of my co-authors, I would like to thank the reviewers for their positive and constructive comments and suggestions on our manuscript entitled “Comparative efficacy of tenofovir and entecavir in nucleos(t)ide analogue-naive chronic hepatitis B: A systematic review and meta-analysis” (PONE-D-19-21194).

We have studied reviewer’s comments carefully and tried our best to revise our manuscript according to the comments. These changes did not influence the main content of the paper. We do not list the changes here; rather, the changes are marked in the revised paper using the track changes feature of Microsoft Word. We are submitting the revised manuscript and revised Tables, and the responses to the comments are listed below point by point.

We would like to express our great appreciation to reviewers for their comments on our paper. We hope that it is acceptable for publication in the journal. If you have any questions about this paper, please do not hesitate to let me know.

Looking forward to hearing from you soon.

Sincerely

Mr. Chen

A point-by-point response

Reviewer #1: There is only minor comment. Since entecavir was prescribed earlier compared to tenofovir, authors should perform a subgroup analysis focusing studies with similar follow-up treatment duration.

Response: We highly appreciate your valuable comments. We performed subgroup analyses for different follow-up times. Accordingly, the abstract, results, and discussion have been revised. These changes did not influence the main content of the paper.

Reviewer #2: 1. There are few new information in the present manuscript. The main goals of antiviral therapy in chronic hepatitis B patients are decreasing liver related mortality and morbidity by suppressing viral replication. Therefore, the primary goal of the manuscript should be focused in achieving these goals, not the percentage of ALT normalization nor HBV reduction, which are already well-known. Comparing the antiviral effect between entecavir and tenofovir cannot make any significant new finding in the clinical aspect.

Response: Thank you for this suggestion. The article is a meta-analysis, which is a quadratic analysis of the literature. Although we would have liked to introduce novel techniques and methods, adopting such novel techniques and methods is difficult if corresponding studies have not been performed or only a single study is available in the literature. Generally, more data can be obtained by adopting more widely used techniques or methods, and more data can make the results of the meta-analysis more reliable. Very novel techniques and methods that have only been reported in one study would not be of significance for a meta-analysis. We will monitor such research and update the meta-analysis if more novel articles are published. These details will be generalized in the limitations section of this study.

Reviewer #3: This study (PONE-D-19-21194) was a systemic review and meta-analysis to compare effectiveness of tenofovir and entecavir.

1. The authors suggested that tenofovir was more effective than entecavir in inhibiting the virus in the early stage, while entecavir was more effective than tenofovir in protecting liver function in the early stage. However, we all know that improvement of liver function follows suppression of HBV replication. Therefore, please discuss why early HBV suppression by tenofovir could not improve the liver function in the early stage.

2. This study compared the HBV DNA suppression and ALT normalization between entecavir and tenofovir. However, there are discrepancies in the definitions of HBV DNA suppression and ALT normalization among studies as follows. How the authors treat these discrepancies in their analysis?

Ref No	*ULN of ALT*	*Lower limit of HBV DNA detection*
8	*40 IU/L*	*20 IU/mL*
9	*31 IU/L*	*2.1 log10 copies/mL*
10	*Not mentioned*	*20 IU/mL*
11	*Journal search failed*	*Journal search failed*
12	*Not mentioned*	*Not mentioned*

3. One study (Ref No 11) could be searched only by "China Knowledge Resource Integrated Database". Therefore, this study could not be reviewed by all peoples except Chinese. I think this study should be excluded in the meta-analysis to improve the clearness of the study.

Response: Thank you for these suggestions.

Why tenofovir is better at suppressing the virus in the early stages of CHB but less protective of liver function than entecavir will be mentioned in the discussion. Few specific basic studies have focused on the differences between oral TDF and ETV,

and the answer can only be found through clinical practice and a survey of the literature. First, both ETV and TDF have strong antiviral and liver function improvement effects, and the possible reasons for these effects include the following: (1) a high immune response can damage liver cells but also suppresses the hepatitis B virus; (2) drug metabolites damage liver cells, and ETV and TDF are antiviral drugs and not liver-protecting drugs; thus, although most of their metabolites are excreted through the kidneys, they all target the liver and may have different effects on the liver while clearing the virus; (3) Bias or other reasons may cause deviations, and because meta-analyses collate scattered data for analysis, small effects may be magnified to produce meaningful results. These possibilities are hypothetical; however, if the reasons for these effects can be worked out, antiviral drugs can be effective in protecting liver function while providing high antiviral activity.

Reviewer #3 provided a careful review and identified discrepancies in the definitions. The meta-analysis required that the included studies be similar but also present differences that could not be absolutely unified. For example, different analyzers define the normal value of ALT differently and different analytical methods measure different HBV thresholds. Therefore, the normal amount of ALT was not limited. We believe that the use of different ALT values in the literature used in the meta-analysis will not affect the reliability of this study. The conversion between units of HBV-DNA measurement was as follows: 1 IU/mL is approximately equal to 5-6 copies/mL. Therefore, the lower limit of quantitation (LLQ) of each experiment is similar. These heterogeneities between studies can be discussed in this meta-analysis.

Hou-xiong Lin's study is in Chinese. In the Methods section of our study, we stated that Chinese literature and English literature will be included. We agree that there are many low-quality papers in the Chinese literature. When performing research, we usually do not search the CNKI database because the quality of some research is unacceptable. However, China has more than one hundred million chronic hepatitis b carriers. Chinese researchers attach great importance to the study of CHB and the level of research on CHB in China is acceptable. Therefore, we do not believe

that this research should be left out. The basic requirement of the meta-analysis is to be comprehensive, so this information will be added to the discussion. The reliability of the research will be further analyzed; moreover, we will remove this study and determine whether the results are affected.
